# The lifetime healthcare costs of female obesity: modeling of England data and the costs of current pharmacotherapy

**DOI:** 10.1097/XCE.0000000000000310

**Published:** 2024-09-20

**Authors:** Adrian H. Heald, Mike Stedman, John Warner-Levy, Martin B. Whyte, Martin K. Rutter, John Martin Gibson

**Affiliations:** a The School of Medicine and Manchester Academic Health Sciences Centre, University of Manchester, Manchester; b Department of Diabetes and Endocrinology, Salford Royal NHS Foundation Trust, Salford; c RES Consortium, andover; d Department of Clinical and Experimental Medicine, University of Surrey, Guildford; e Division of Diabetes, Endocrinology and Gastroenterology, School of Medical Sciences, University of Manchester, Manchester; f Diabetes, Endocrinology and Metabolism Centre, Manchester University NHS Foundation Trust; g Manchester Academic Health Science Centre, NIHR Manchester Biomedical Research Centre and

Obesity has major impacts on all-cause mortality risk, healthcare resource utilization (HCRU), [1] and quality of life [2]. It is associated with an increased risk of developing long-term conditions such as type 2 diabetes, cardiovascular disease, hypertension, and a number of cancers, while also being associated with premature death [3]. In women, obesity can adversely affect reproductive health, leading to obstetric and neonatal complications, as well as underpinning the development of breast and endometrial cancers [4].

Glucagon-like peptide-1 (GLP-1) receptor agonists and associated incretin therapies have been shown to lower body weight, improve cardiovascular outcomes, and lower blood glucose levels [5]. However, the affordability of these medications remains a concern for the National Health Service in the UK and elsewhere, given the constraints of limited resources. It is therefore important to estimate the lifetime health costs associated with obesity, as this will inform decision-making regarding the use of drugs such as GLP-1 agonists.

The numbers of men and women with Class I/II obesity in England are similar, but Class III obesity is twice as prevalent in women than men [6,7]—nearly 600 000 women under 50 years of age have Class III obesity [7]. We have here estimated the lifetime costs of medical care for women with Class I/II obesity and for Class III obesity, compared to those with a BMI of <25 kg/m^2^.

Regarding available pharmacotherapy to facilitate weight reduction (as distinct from lifestyle intervention programs), in the STEP 1 trial of adults, without diabetes, after 68 weeks 86.4% of the GLP-1 (semaglutide) group lost at least 5% on baseline weight, compared with 31.5% for placebo. Semaglutide led to 69.1% (vs. 12.0%) losing ≥10% weight (69.1 vs. 12.0%), and ≥20% reduction of baseline weight was achieved in 32.0 (vs. 1.7%) [8]. The SURMOUNT-1 trial found that in people with overweight/obese but without diabetes, 72 weeks of the combined GLP-1/glucose-dependent insulinotropic peptide (GIP) agonist, tirzepatide, at doses of 5, 10, or 15 mg led to 15.0, 19.5, and 20.9% weight loss, respectively, compared with 3.1% in people taking placebo [9]. For the co-primary endpoint of the proportion of people attaining at least a 5% reduction in their baseline bodyweight, the corresponding values were 85%, 89%, and 91% vs. 35% for placebo.

When examining the transition between established BMI categories, in the global medical literature, the proportions of trial entrants transitioning across BMI categories are not well reported, and so projections concerning transition between BMI categories are very challenging. While significant weight loss is achieved, many individuals with obesity or overweight are likely to require additional therapies if normalization of BMI to <25 kg/m^2^ was the goal [10].

In this context, we examined the female population in England split by age and BMI class, based on published data. Consequent life expectancy and HCRU costs (HCRU) [11] which include costs of treatment and hospitalization were calculated for each BMI class and compared to those with a BMI of <25 kg/m^2^. The relative health cost for individuals by BMI category was not reliably available for males, hence the female only focus here. The total HCRU (based on current costs) was added up over the future expected life years to give a total Lifetime HCRU (LT-HCRU). The association with obesity was then estimated both in total and per person, with adjustment for the loss of life years. As the increased HCRU due to BMI will occur at older age, lifetime costs capture this effect. However, the increased mortality and corresponding reduction in expected life years can offset this increase.

Table 1 shows the expected excess lifetime health costs at population level and per person vs. sex and age-matched individuals with BMI < 25 kg/m^2^. Derived from Table 1, females with BMI 30–39.9 kg/m^2^, representing 29% of the female adult population, lose 17.5 million expected life years with a net additional LT-HCRU of £9481/person, while females with BMI ≥ 40; 4.3% of this population, lose 6.1 million expected life years with additional LT_HCRU of +£8565/person; of these, the 2.6% aged 16–49 years lose 1 million expected life years with a net additional LT-HCRU costs of +£10 736/person.

**Table 1:** Nominal expected excess lifetime health costs at a population level and per person vs sex and age-matched Individuals with BMI < 25 kg/m^2^

	Population, 000	Average age (years)	Life expectancy (years)	Total lifetime HCRU (£ billion) summated for the population	Lifetime HCRU/person
Overall	23 180	48.8	37.4	£2255	£97 322
BMI 30–39.9 kg/m^2^	6744	51.6	32.7	£671	£99 520
Difference to BMI < 25 kg/m^2^			−2.6	+£63	+£9481
BMI ≥ 40 kg/m^2^	1101	46.3	33.8	£106	£105 445
Difference to BMI < 25 kg/m^2^			−6.1	+£8.7	+£8565
BMI ≥ 40 kg/m^2^ & age 16–49 years	595	34.9	43.4	£71	£120 605
Difference to BMI < 25 kg/m^2^			−7.0	+£6.3	+£10 736

HCRU, healthcare resource utilization.

In Figure 1 we have provided a breakdown by age band and BMI category for future life years lost per person and additional lifetime HCRU per person.

**Fig. 1:**
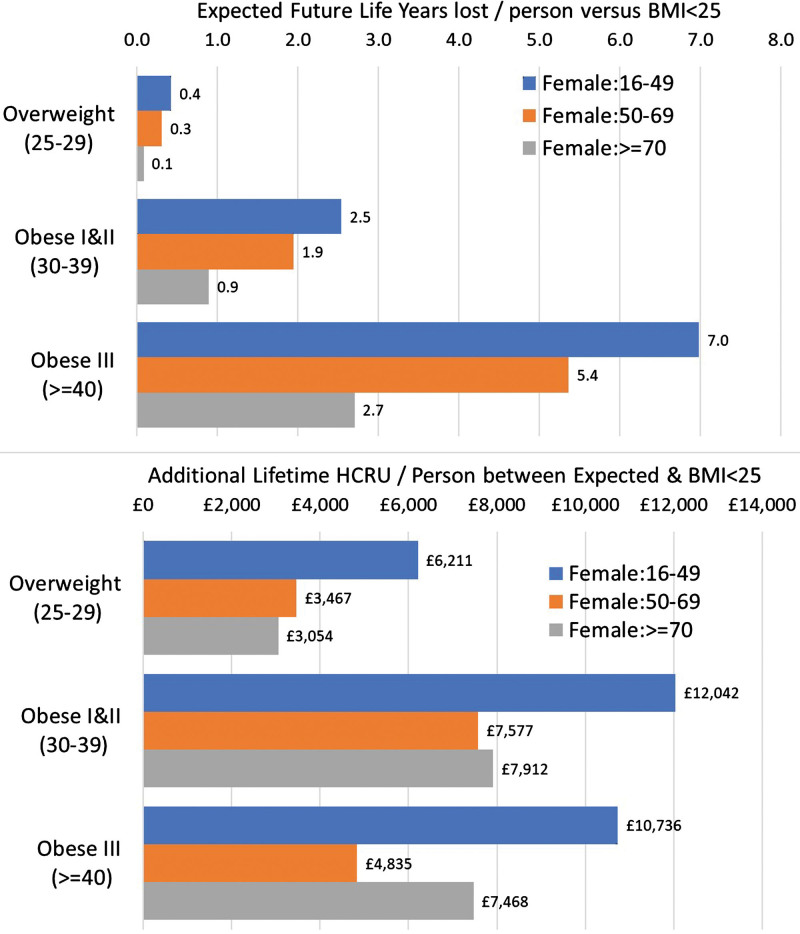
Analysis of future life years lost and additional lifetime HCRU per person by BMI category and age band. BMI, body mass index; HCRU, healthcare resource utilization.

It has been reported that younger women are at the highest risk of being overweight [6,7]. Weight control programs, including, for example, a very low-calorie diet, are expensive and medication support such as GLP-1 agonists costs up to £1200/€1400 per year [12], with the long-term sustainability of both these approaches unpredictable at an individual level [10].

In the case of women with obesity, the direct estimated lifetime HCRU gains, as reported above, may not justify investment in weight-reducing pharmacotherapy. However, if 50% of life years lost could be avoided and a value of £30 000 associated with each life year gained, then the additional benefits for BMI ≥ 30 kg/m^2^ could be around £40 000/person; BMI ≥ 40 kg/m^2^ £90 000/person; and of those with BMI ≥ 40 kg/m2 and aged 16–49 years: £105 000/person.

In the light of this analysis, we suggest that the construction of financial cases for GLP-1 or other incretin therapy initiation and other treatment options over many years must also take into account the potential improvements in a person’s quality of life and improved survival, in order to provide sufficient evidence from a health economic perspective, for the benefit of these pharmacological interventions in the longer term.

## Acknowledgements

M.S. undertook data assembly and data analysis with the help of J.W.-L. A.H. and M.S. conceived the study. J.W.-L. assisted with the collation of references as did M.B.W. M.B.W., J.M.G. and M.K.R. assisted with the interpretation of results. All authors were involved in interpreting the findings reviewing/editing of the manuscript.

The datasets analyzed during the current study are publicly available to anyone who wishes to access them.

As this study utilized already published publicly available or aggregated data for analysis, it was not felt that ethical approval was required.

### Conflicts of interest

There are no conflicts of interest.
